# Probabilistic coverage of the frontal aslant tract in young adults: Insights into individual variability, lateralization, and language functions

**DOI:** 10.1002/hbm.26630

**Published:** 2024-02-20

**Authors:** Wen‐Jieh Linn, Jessica Barrios‐Martinez, David Fernandes‐Cabral, Timothée Jacquesson, Maximiliano Nuñez, Ricardo Gomez, Yury Anania, Juan Fernandez‐Miranda, Fang‐Cheng Yeh

**Affiliations:** ^1^ Department of Neurological Surgery University of Pittsburgh Pittsburgh Pennsylvania USA; ^2^ CHU de Lyon – Hôpital Neurologique et Neurochirurgical Pierre Wertheimer Lyon France; ^3^ Department of Neurological Surgery Hospital El Cruce Buenos Aires Argentina; ^4^ Department of Neurological Surgery Stanford University Stanford California USA; ^5^ Department of Bioengineering University of Pittsburgh Pittsburgh Pennsylvania USA

**Keywords:** frontal aslant tract, population differences, tractography

## Abstract

The frontal aslant tract (FAT) is a crucial neural pathway of language and speech, but little is known about its connectivity and segmentation differences across populations. In this study, we investigate the probabilistic coverage of the FAT in a large sample of 1065 young adults. Our primary goal was to reveal individual variability and lateralization of FAT and its structure–function correlations in language processing. The study utilized diffusion MRI data from 1065 subjects obtained from the Human Connectome Project. Automated tractography using DSI Studio software was employed to map white matter bundles, and the results were examined to study the population variation of the FAT. Additionally, anatomical dissections were performed to validate the fiber tracking results. The tract‐to‐region connectome, based on Human Connectome Project‐MMP parcellations, was utilized to provide population probability of the tract‐to‐region connections. Our results showed that the left anterior FAT exhibited the most substantial individual differences, particularly in the superior and middle frontal gyrus, with greater variability in the superior than the inferior region. Furthermore, we found left lateralization in FAT, with a greater difference in coverage in the inferior and posterior portions. Additionally, our analysis revealed a significant positive correlation between the left FAT inferior coverage area and the performance on the oral reading recognition (*p* = .016) and picture vocabulary (*p* = .0026) tests. In comparison, fractional anisotropy of the right FAT exhibited marginal significance in its correlation (*p* = .056) with Picture Vocabulary Test. Our findings, combined with the connectivity patterns of the FAT, allowed us to segment its structure into anterior and posterior segments. We found significant variability in FAT coverage among individuals, with left lateralization observed in both macroscopic shape measures and microscopic diffusion metrics. Our findings also suggested a potential link between the size of the left FAT's inferior coverage area and language function tests. These results enhance our understanding of the FAT's role in brain connectivity and its potential implications for language and executive functions.

## Graphical abstract

The study revealed individual differences in the left anterior frontal aslant tract (FAT), especially in the superior region, with significant left lateralization. Functional analysis reveals a correlation between the left inferior coverage area and language function. Overall, it provides valuable insights into FAT connectivity and its role in language processing.

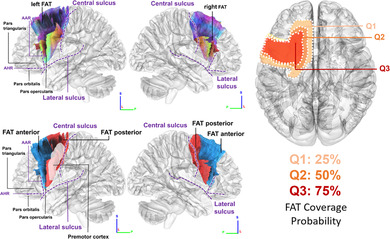

**Practitioner Points**

Although previous studies have shed light on the structure and function of the frontal aslant tract (FAT), a population‐based investigation of individual differences in FAT remains to be conducted, which is a critical factor in determining the distribution and segmentation of the pathway. The study included 1065 young adults from the Human Connectome Project, using automated tractography and cadaveric dissection validation to map the distribution patterns of the FAT neural pathway. Advanced shape analysis techniques were applied to investigate the relationship between FAT morphology and language processing abilities, providing insights into the neuroanatomical basis of language and potential clinical applications.

## 1 INTRODUCTION

The frontal aslant tract (FAT) is described as related to language function recently and has drawn a lot of attention (Chernoff et al., 2018; Dick et al., 2019). Studies have been conducted to identify and reveal the structure of FAT. Aron et al. (2007) first proposed a connection between the inferior frontal cortex (IFC) and the pre‐supplementary motor area (pre‐SMA), while Lawes et al. (2008) reported a fiber track extending from the superior frontal gyrus to the pars triangularis. Although the tract was reported in these studies, it was not formally named until Catani et al. (2012) used diffusion imaging tractography and dissection to give the fiber tract its name as FAT. They showed that FAT extended from the dorsal and medial cortex of the superior frontal gyrus (SFG) to the posterior region of the inferior frontal gyrus (IFG). In some cases, the FAT projected into the pars triangularis of the IFG and the inferior region of the precentral gyrus (PrCG). The oblique geometry of FAT accounts for its name *aslant*. Briggs et al. (2020) further investigated the white matters related to SFG, and characterized the FAT as a curved fiber that gradually bends approximately 90 degrees before terminating in the pars opercularis and pars triangularis. They highlighted that the FAT extended deeply into the superior longitudinal fasciculus (SLF), which ran orthogonally to the FAT while they are orthogonal to the FAT in the anterior–posterior plane. This underscored the significance of removing the SLF carefully to expose the FAT when operating dissection.

Besides studying the structure of the FAT, Bozkurt et al. (2016) looked at how the FAT connects with nearby pathways and examined the spatial positioning of the FAT alongside pathways such as the SLF, arcuate fasciculus (AF), callosal fibers, corticospinal tract (CST), and frontostriatal tract. Notably, they observed that the FAT traversed the superior insular, limiting sulcus to reach the IFG, in contrast to corona radiata fibers, which run beneath the superior insular, limiting sulcus to terminate in the central core.

Additionally, lots of studies indicated that FAT, joint with other associated pathways, facilitates language related, motor planning, and executive functions. In a subsequent study employing diffusion tractography, Catani et al. (2013) explored the language‐functional aspects of the FAT. They examined the association between the FAT and verbal fluency as well as grammar impairment in patients with primary progressive aphasia. Their findings indicated a stronger association between the FAT and verbal fluency rather than grammar impairment. Another study conducted by Sierpowska et al. (2015) focused on a patient who exhibited a morphological overregularization strategy in a verb generation task during awake brain tumor resection surgery. The findings suggested that damaging the left FAT resulted in a language deficit specifically related to word finding. Similarly, Kinoshita et al. (2015) investigated 19 patients undergoing awake surgery for frontal gliomas, discovering a correlation between the left FAT and speech control. They also noted that the left FAT was not only associated with language fluency but also played a role in both semantic and phonemic processing. Furthermore, Dick et al. (2019) included in their review study that the FAT had potential in involving motor planning and executive functions. Various studies exploring the connection between the FAT and persistent developmental stuttering might suggest that the FAT contribute to the motor control aspects of speech (Dick et al., 2014; Kronfeld‐Duenias et al., 2016; Neef et al., 2018). Dick et al. also highlighted Swann et al.'s (2012) study, which investigated the physiological connectivity between the right pre‐SMA and right IFG, identified as the FAT though not explicitly labeled. This connectivity was observed during stop‐signal trials, suggesting their engagement in inhibitory control tasks as part of executive functioning. Additionally, in a study demonstrating a unified model of post‐stroke language deficits, the FAT is identified as being associated with verbal quantity and executive functions (Alyahya et al., 2020). Overall, most studies consistently showed that the FAT plays a crucial role in both language processing and executive control.

Although previous studies have shed light on the structure and function of the FAT, a population‐based investigation of *individual differences* in FAT remains to be conducted. While several studies have suggested that the pars opercularis is the primary source of connectivity in the FAT (Catani et al., 2012; Szmuda et al., 2017), other studies have shown that in some cases, the pars triangularis may be the primary contributor of fibers (Marian‐Magaña et al., 2022). These discrepancies suggest that there could be substantial individual variation in the distribution of FAT fibers. Given that individual variability is a critical factor in determining the distribution and segmentation of the pathway, it is important to investigate individual differences in FAT. Furthermore, there is a need for a study to explore the relationship between language function and the structural morphology of the FAT. Such a study would require advanced white matter mapping and associated shape analysis on a large cohort.

In this study, we conducted an extensive investigation into the individual variability of the FAT using automatic tractography. Our cohort consisted of 1065 young adults from the Human Connectome Project (HCP), providing a robust sample size for our analyses. To accurately map the FAT bundles, we employed a state‐of‐the‐art automated tractography pipeline (Yeh, 2022). The tract‐to‐region connectome employed trajectory‐based recognition and refrained from filtering the tractogram based on brain regions in order to mitigate circular analysis. We took special care to validate the accuracy of our automated fiber tracking pipeline by comparing the results with cadaveric dissection data, ensuring the reliability of our findings. In order to capture the distribution patterns of the FAT within the population, we aggregated the tractography data, allowing us to calculate the population probability of the FAT connections. By quantifying the likelihood of FAT presence and its connectivity patterns, we gained valuable insights into the prevalence and variations of this important neural pathway among individuals.

To delve deeper into the relationship between FAT morphology and its function, we employed advanced shape analysis techniques (Yeh, 2020). By applying these techniques to our tractography data, we were able to explore potential structure–function correlations between the shape characteristics of the FAT and performance on standardized functional tests in the NIH toolbox (Hodes et al., 2013). From the dataset acquired from the HCP, we selected all the functional tests related to language and executive function. Namely, the Oral Reading Recognition Test and Picture Vocabulary Test were chosen for language function, while the Dimensional Change Card Sort Test and Flanker Inhibitory Control and Attention test were selected for executive function. This approach allowed us to investigate whether specific structural features of the FAT were associated with variations in language and executive abilities, providing insights into the neural mechanisms that associated with these tests.

Our comprehensive study design, combining population‐based tractography, validation through cadaveric dissection, and shape analysis, aimed to provide a thorough understanding of the individual variability of the FAT and its potential implications for language processing. By elucidating the intricate relationship between FAT morphology and language abilities, we contribute to the growing body of knowledge on the neuroanatomical basis of language, paving the way for future research and potential clinical applications.

## 2 MATERIALS AND METHODS

### 2.1 MRI acquisition

We utilized a dataset consisting of 1065 subjects, which was obtained from the HCP and made available by the WashU consortium (Glasser et al., 2016). The diffusion data were acquired using a multishell scheme, incorporating three *b*‐values (1000, 2000, and 3000 s/mm^2^), with 90 directions in each shell. The spatial resolution of the acquired images was 1.25 mm isotropic. The ac–pc line of the ICBM152 space is the standard of alignment. The diffusion data and b‐table were linearly rotated and interpolated with cubic spline interpolation at 1 mm. Q‐sampling imaging with a diffusion sampling length ratio of 1.7 is used to reconstruct the rotated data, which were later used in automated tractography (Yeh et al., 2010). To ensure the accuracy and orientation of the b‐table, an automatic quality control routine (Schilling et al., 2019) was employed.

### 2.2 Behavioral tests

To study the functional correlation of the FAT, we selected tests assessing language function and executive function from HCP's behaviors tests battery. The tests accessing language function included Oral Reading Recognition Test and Picture Vocabulary Test. Specifically, in the Oral Reading Recognition Test, participants read aloud words presented in the test, while in the Picture Vocabulary Test, participants listened to a vocabulary and selected the corresponding picture from a set of four options. The tests accessing executive function included Dimensional Change Card Sort Test and Flanker Inhibitory Control and Attention test. In the Dimensional Change Card Sort Test, participants matched test pictures to two target pictures, initially based on one dimension (e.g., color) and later, after a series of trials, based on another dimension (e.g., shape). In the Flanker Inhibitory Control and Attention test, participants identified the direction in which the middle fish in a row is pointing. We used the uncorrected scores and transformed into Z‐scores for correlation analysis.

### 2.3 Automated tractography

The automated tractography pipeline in DSI studio (http://dsi-studio.labsolver.org) is used to map the 52 white matter bundles of each subject. This pipeline is a combination of deterministic fiber tracking algorithm (Yeh et al., 2013), topology‐informed pruning (Yeh et al., 2019), and randomized parameter saturation (Yeh, 2020), with trajectory‐based tract recognition (Yeh, 2020) being an integrated interface, as detailed in a recent study (Yeh, 2022). Following the mapping process, the white matter bundles of the 1065 subjects were exported to the ICBM152 2009 nonlinear space to examine the population variation of the FAT. The analysis was conducted in DSI Studio package, which is publicly available at http://dsi-studio.labsolver.org.

### 2.4 Dissections

The human brain of five neurological healthy subjects were studied and compared with the results of the fiber tracking done by not only the manual processing but the automatic processing as well. Dissections were performed by expert neurosurgeons (TJ, MN, RG, and YA). Human brains were fixed in 10% formalin for 28 days and frozen for 14 days. Dissections were carefully performed from superficial to deep, starting at the grey matter until white matter fibers were encountered. The anatomical dissections of the FAT were placed in a high degree of difficulty. This is because the fibers of this tract are highly anatomically related to the fibers of the SLF tract, which crosses the bundle and merges in a complex way. The separation of the fibers was done in a way to preserve the integrity of the FAT without destroying the underlying or overlapping tissue.

## 3 RESULTS

### 3.1 Anatomy

**FIGURE 1:**
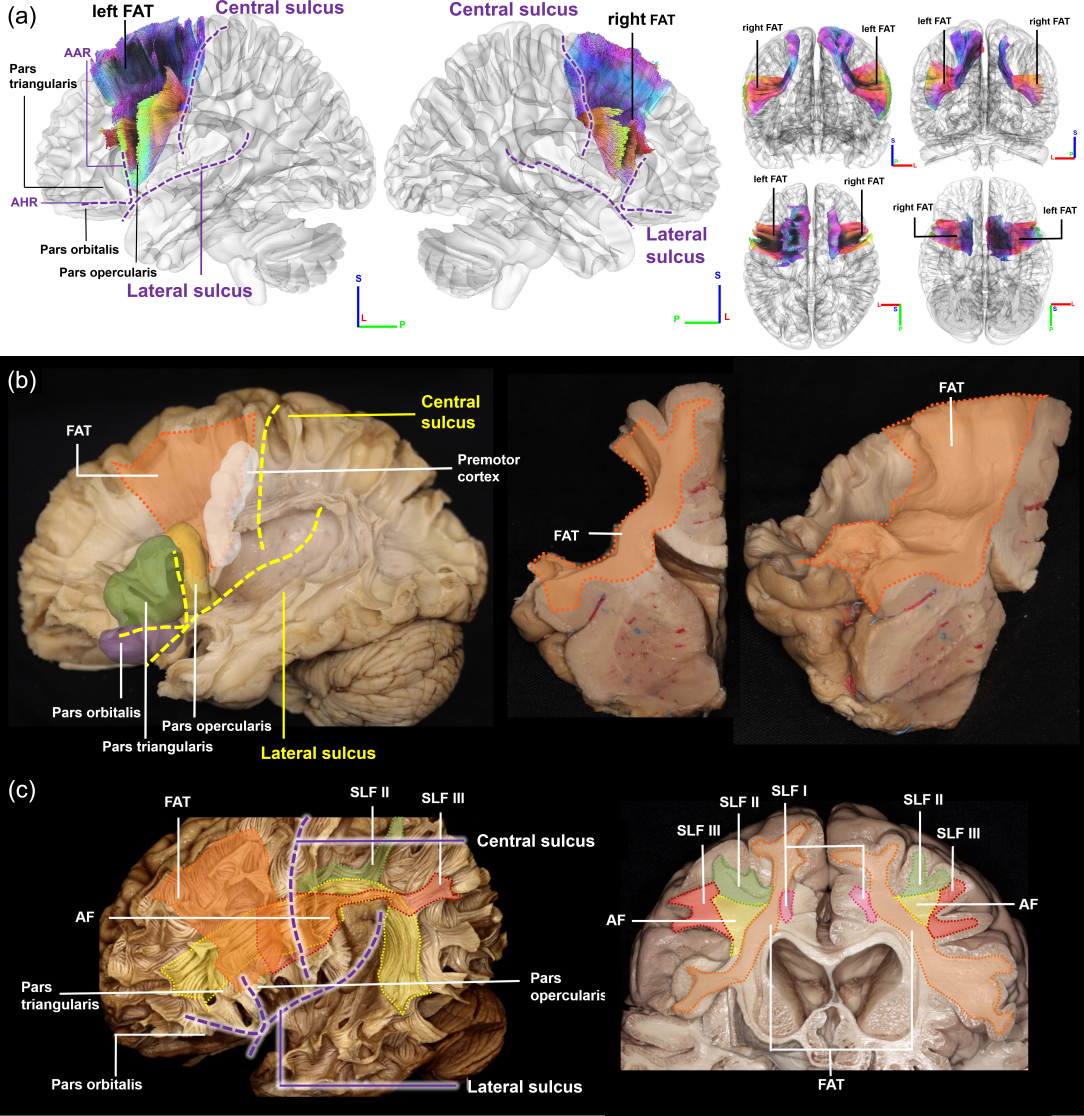
Population‐averaged frontal aslant tract (FAT) and its spatial relations to cortical regions, sulci, and surrounding white matter fiber pathways. (a) The location of FAT is in the frontal lobe, anterior to central sulcus, posterior to anterior ascending ramus (AAR), superior to lateral sulcus, anterior horizontal ramus (AHR), and pars orbitalis. It projects from the premotor cortex to pars opercularis. (b) FAT can be visualized in the white matter cortex specimen (medial frontal gyrus has been removed to facilitate visualization). The coronal view of the FAT shows the white matter fibers running from the superior frontal gyrus (SFG) to the inferior frontal gyrus (IFG). Lateral view of the FAT visualizes the fiber tract connecting the pre‐supplementary and supplementary motor cortex areas to the inferior frontal gyrus. (c) FAT and other tracts are color coded and visualized in the white matter cortex specimen. The location of FAT runs more medial to other surrounding structures, such as superior longitudinal fasciculus (SLF) II, SLF III and arcuate fasciculus (AF), whereas SLF I is medial to the FAT.

We first examined the population‐averaged atlas of the FAT (Yeh et al., 2018) and its relationship with surrounding pathways. As shown in Figure 1a, FAT is situated in the frontal lobe, anterior to central sulcus, posterior to anterior ascending ramus, superior to lateral sulcus and anterior horizontal ramus. The FAT pathways connect the superior frontal regions, premotor cortex, and pars opercularis. The right FAT has similar geometry and the population‐average atlas demonstrates lateralization, which was later examined. The FAT is depicted in orange in the white matter cortex specimen in Figure 1b, projecting through pars opercularis (yellow), pars triangularis (green), and premotor cortex (white). In Figure 1c, an anatomy dissection image from another subject demonstrates the relationship of FAT and its surrounding white matter fiber pathways. FAT is positioned more medially to the SLF II (green), SLF III (red) and AF (yellow), while the SLF I (pink) is medial to the FAT. The SLF I, SLF II, and SLF III runs in the orthogonal direction to the FAT. The anterior part of the AF also runs in the orthogonal direction to the FAT, but its posterior part has a curve of approximately 90° and becomes parallel to the orientation of the FAT.

### 3.2 Population probabilities

**FIGURE 2:**
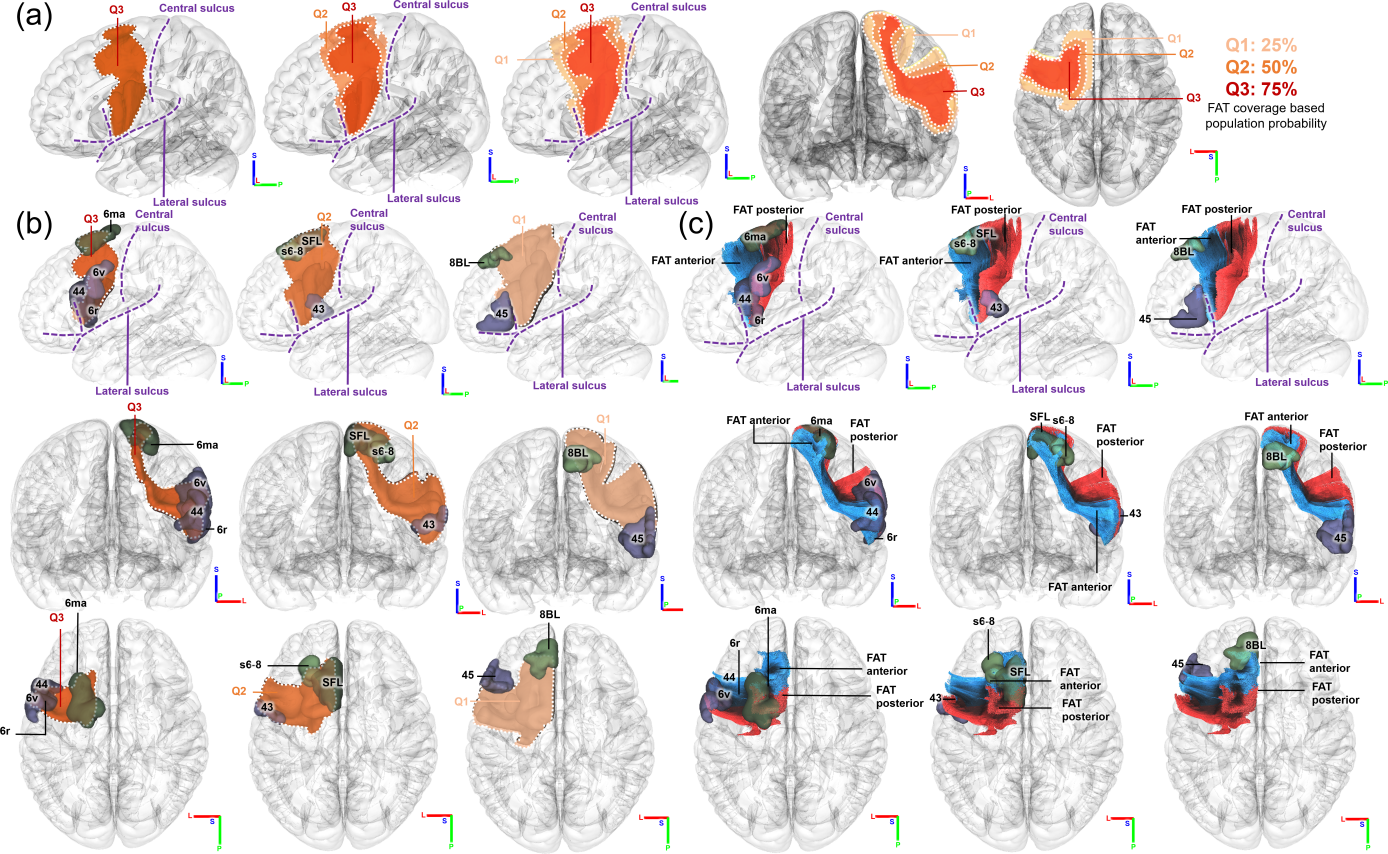
Population probabilities of frontal aslant tract (FAT) coverage at first, second, and third quantiles (Q1, Q2, Q3) of the human young adults. (a) Q2 has 119.53% more coverage in volume than Q3, whereas Q1 has 66.97% more coverage volume than Q2. There are substantial individual differences in FAT coverage in the young adult population, particularly at the anterior portion of FAT. (b) Q3 mainly covers area 6ma, 44, 6r, and 6v in Human Connectome Project (HCP)‐MMP atlas. Besides the areas that Q1 covers, Q2 also covers s6‐8, superior longitudinal fasciculus (SLF), and 43. In addition to the areas that Q3 and Q2 cover, Q1 covers 8BL and 45 as well. (c) Among all the areas Q3 covers, 6ma is traversed by both posterior and anterior FAT, while 6r and 6v is traversed by the posterior FAT, 44 innervates the anterior FAT. For the Q2 coverage areas, SFL innervates both posterior and anterior FAT, while 43 innervates the posterior FAT, s6‐8 innervates the anterior FAT. Within the areas Q1 mainly covers, both 8BL and 45 are traversed by the anterior FAT.

We conducted a probabilistic analysis of individual differences in the coverage of the FAT among 1065 young adults. Figure 2 displays the population probabilities of FAT coverage at the first, second, and third quantiles (Q1, Q2, Q3), where darker colors indicate higher coverage percentages. Our findings reveal a significant variability in FAT coverage, particularly in the superior frontal and middle frontal gyrus, with larger differences observed in the superior portion. Additionally, we observed relatively minor variations in the FAT regions proximal to the central sulcus. We quantified the volume of Q3, Q2, and Q1, with volumes of 8932; 19,608; and 32,740 m^3^, respectively. Q3 represents FAT coverage that were shared between more than 75% of the population, whereas Q2 and Q1 represent those shared between 50 and 25% population, respectively. Q2 has 119.53% more coverage volume than Q3, while Q1 has 66.97% more coverage volume than Q2, suggesting that there are substantial individual differences, mostly at the anterior portion of the FAT.

We further used the HCP‐MMP atlas to identify cortical regions. As shown in Figure 2b,c, our results show that Q3 covers regions 6ma (the lateral aspect of the most superior part of the superior frontal gyrus), 44 (pars opercularis), 6r (the anterior aspect of the most inferior part of the PrCG), and 6v (the lateral aspect of the PrCG, located 1/3 from its most inferior part). This suggests that the majority of population has these regions run through by FAT. We roughly segment the FAT into anterior and posterior parts based on the areas it runs through. Both parts run through 6ma, whereas only the posterior FAT runs through 6r and 6v, and only the anterior FAT runs through 44. Furthermore, Q2 covers s6‐8 (the lateral aspect of the superior frontal gyrus, positioned a quarter posterior from its middle portion), SFL (the medial aspect of the most superior part of the superior frontal gyrus), and 43 (the lateral aspect of the most inferior part of the PrCG), with SFL run through by both posterior and anterior FAT, while 43 run through only by the posterior FAT, and s6‐8 run through only by the anterior FAT. Additionally, Q1 covers 8BL (the medial aspect of the middle portion of the superior frontal gyrus) and 45 (pars triangularis), in addition to the regions covered by Q3 and Q2. Both regions are run through by the anterior FAT, suggesting that most individual differences are located at anterior FAT around 45 and 8BL.

**FIGURE 3:**
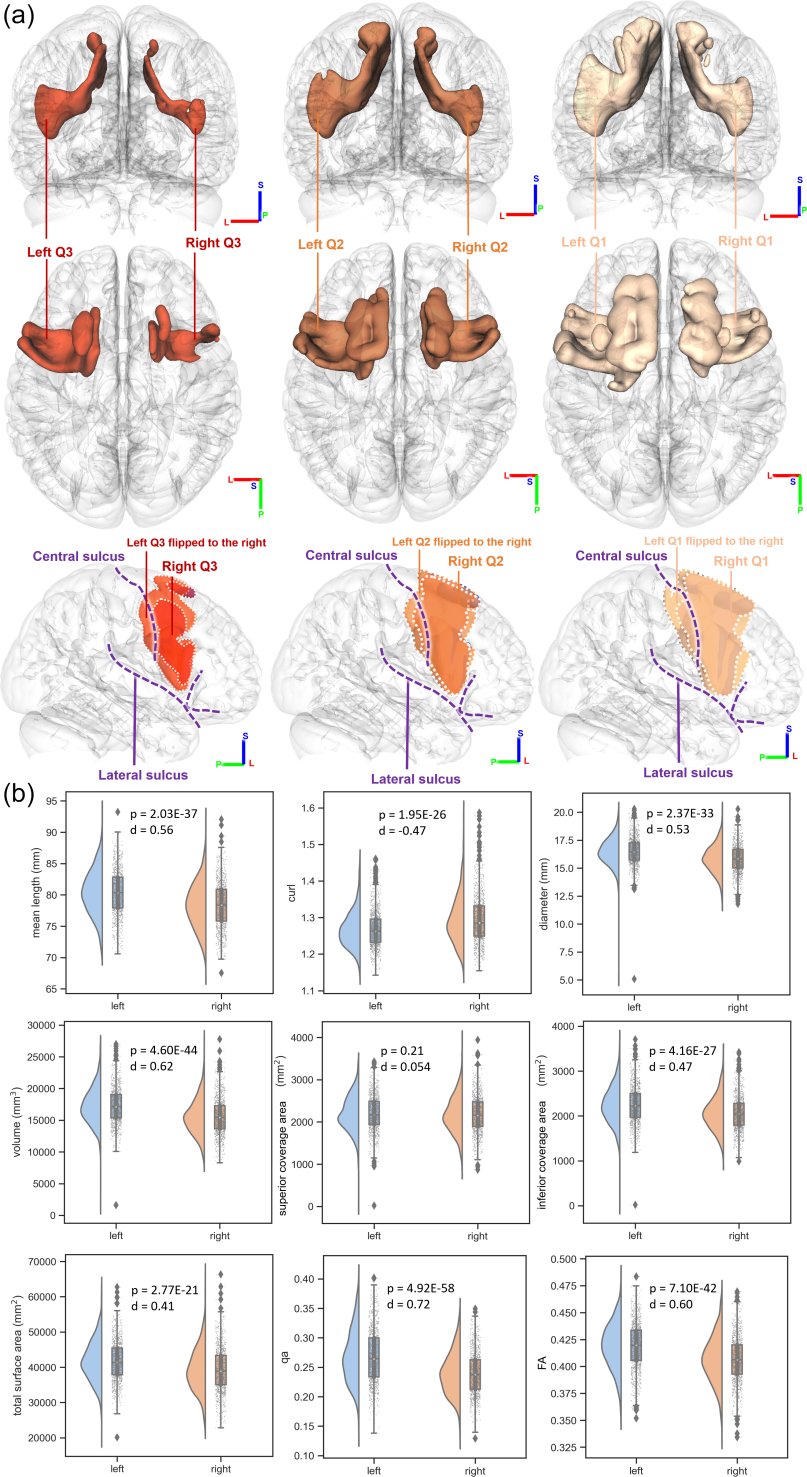
Comparison between the left and right frontal aslant tract (FAT). (a) The population probabilities of FAT coverage shows that the left FAT has a larger individual difference compared with the right FAT. The first two rows show that the left FAT has a larger volume in all three quantiles, while the third row mirrors the left FAT to the right side to facilitate comparison. The results shows that the left side exhibits a greater degree of individual variability. More prominent disparities in individual differences between the left and right FAT arise in the inferior and posterior coverage area. (b) The statistics comparisons of the left and right FAT and their Cohen's *d* showing lateralization. Although most of the covariates are left biased, the curl of the FAT is right biased. The superior coverage area has the least lateralization, while the QA of the FAT has the most lateralization.

Figure 3a illustrates the lateralization of the FAT coverage based on population probability. The first two rows demonstrate that the left FAT has a larger volume in all three quantiles, while the third row presents a mirrored image of the left FAT on the right side for ease of comparison. Our findings indicate that the left FAT exhibits a greater degree of individual variability than its right counterpart. Furthermore, more individual differences have emerged in the inferior and posterior coverage area, highlighting significant disparities between the left and right FAT.

Figure 3b further compares shape and diffusion metrics between the left and right FAT in 1065 young adults. The superior coverage area did not display a significant difference (*p* = .21), along with a small effect size (*d* = 0.054), while all remaining metrics showed strong significant differences (*p* < .001) with medium effect sizes (Cohen's *d* ranged from 0.4 to 0.7). All metrics demonstrated left dominance, with the exception of curl, which exhibited a more curved structure in the right FAT. Notably, there were distinct differences in the inferior coverage areas, consistent with the findings in Figure 3a. Among all the statistics, QA exhibited the most significant difference with a Cohen's *d* value of 0.72, suggesting that the left FAT is a more compact fiber bundle. Overall, FAT exhibits significant left lateralization in both macroscopic shape measures and microscopic diffusion metrics.

**FIGURE 4:**
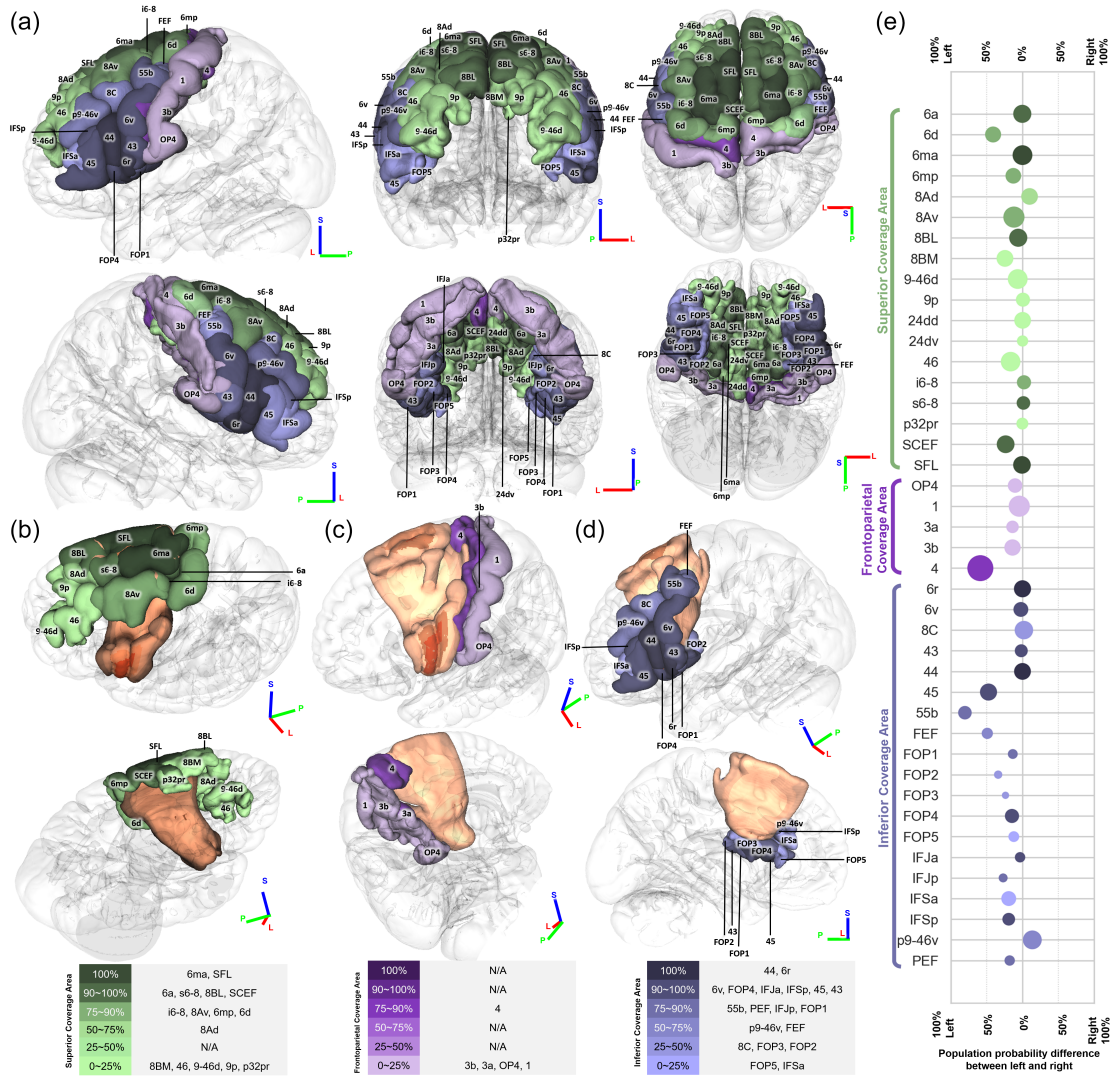
Population probabilities of the frontal aslant tract's (FAT) coverage in cortical regions. (a) Green regions are run through by the superior part of the FAT, while purple regions are run through by the frontoparietal part, and blue regions are run through by the inferior part. The darker color represents higher population probability. (b) The orange area is the FAT with the different shades representing Q1, Q2, and Q3. The FAT shows substantially connection with the areas in the superior frontal gyrus. (c) Other than area 4, other cortical regions have low population probabilities. (d) The areas with high population probabilities are mainly in the inferior frontal gyrus, including the pars opercularis and pars triangularis. (e) The population probability difference shows that the individual difference of most of the coverage areas are left biased. The surface area of the circle indicates the volume of the area.

We presented the population probabilities of the FAT's coverage in various cortical regions and specified its coverage area in Figure 4. These probabilities were calculated based on a tract‐to‐region connectome using the HCP‐MMP parcellations (Yeh, 2022). The cortical regions were color‐coded to indicate different coverage compartments of the FAT bundles and regions. Specifically, green represented superior coverage area, blue represented inferior coverage area, and purple represented frontoparietal coverage area. Figure 4b–d showed brain areas of the left hemisphere, and the probability of each area being ran through by the FAT was quantified. Area 44 (pars opercularis) had a higher probability of being traversed by the inferior FAT over area 45 (pars triangularis), but both had a high population probability of over 90% of being traversed by the FAT, indicating that most people have FAT connections in both areas. Figure 4e showed the population probability difference of the FAT coverage between left and right, with the circle's surface area proportional to the average volume of the area. The colors are coded in the same way. A majority of the regions, 31 in total, were left‐biased in percentage, while the most biased ones including areas FEF (the medial aspect of the middle portion of PrCG), 45 (pars triangularis), 55b (the lateral aspect of the middle portion of PrCG), and 4 (the medial aspect of the posterior part of PrCG). These areas are located in the anterior and posterior parts of the FAT, further supporting the conclusion drawn in Figure 3 that there is a greater degree of individual variability in this region, thereby underscoring the notable disparities between the left and right FAT.

### 3.3 Tract‐to‐region connections

**FIGURE 5:**
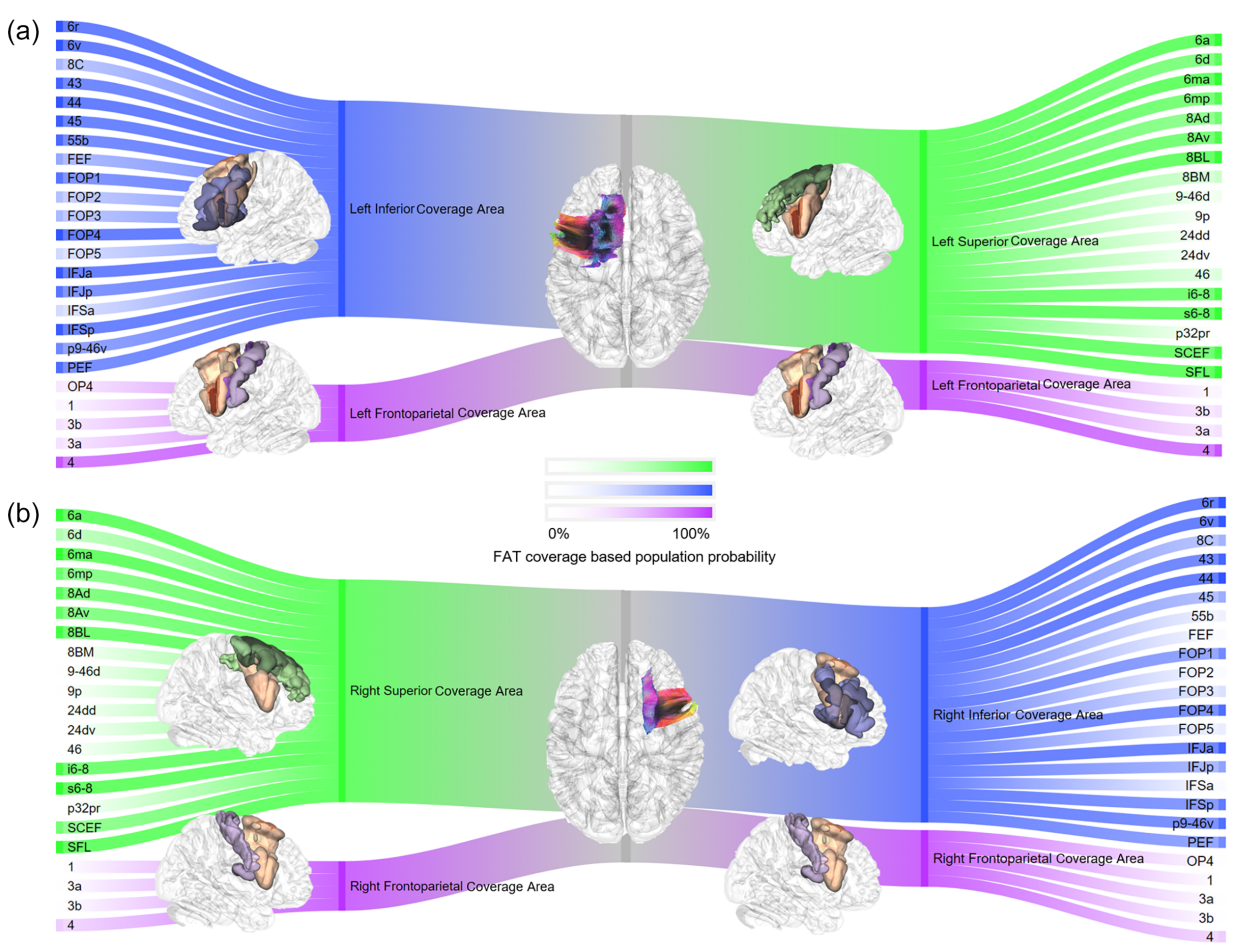
Sankey flow diagram showing the frontal aslant tract's (FAT) coverage in cortical regions. (a) The color saturation represents the population probability of cortical regions. This figure is showing the left FAT and the cortical regions it traverses. (b) This figure is showing the right FAT and the cortical regions it traverses.

The findings pertaining to the connectivity of the FAT are presented in Figure 5, a Sankey flow diagram that portrays the probabilities of the same source population, as previously demonstrated in Figure 4. Figure 5a,b illustrates the connections in the left and right hemispheres, respectively. The saturation of each region's color in the diagram corresponds to its population probability. The colors used in Figure 5 are also utilized to represent the superior, inferior, and frontoparietal coverage of the FAT, in green, blue, and purple, respectively. The diagram reveals that the inferior areas exhibit greater coverage of the FAT than the superior regions.

### 3.4 Structure–function correlation

**FIGURE 6:**
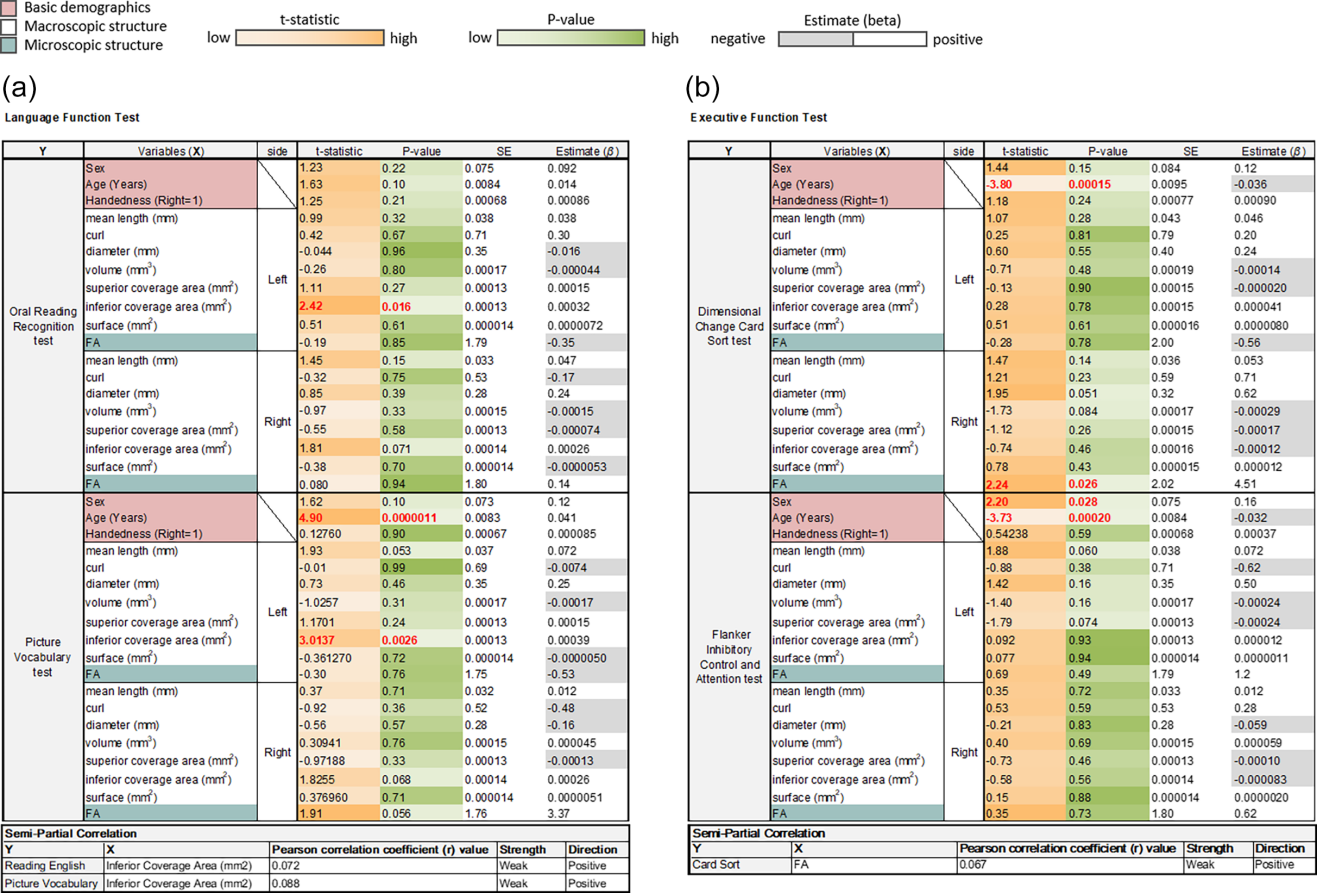
Results of the correlation analysis between the frontal aslant tract (FAT) and function tests. (a) The inferior coverage area of the left FAT shows a significant correlation (*p* = .016) with the Oral Reading Recognition Test. The Picture Vocabulary Test shows a significant correlation with the left FAT's inferior coverage area (*p* = .0026). The Pearson correlation coefficient values are determined through semipartial correlation analysis. The correlations of both tests and the inferior coverage area of the FAT exhibit a weak correlation. (b) The Dimensional Change Card Sort Test score correlated with FA (*p* = .026). The Card Sort Test and FA exhibit a weak correlation.

In Figure 6, we presented the association between the FAT and language function tests, as well as executive function tests. We used a linear fixed‐effect model to regress participants' covariates on the language and executive functions, respectively. During the regression analysis, subject number 114621 was excluded from the dataset due to issues with FAT volume measurement, rendering it an outlier.

Figure 6a shows the correlations with the two language function tests, respectively. The inferior coverage area of the left FAT exhibited a significant positive correlation (*p* = .016) with the Oral Reading Recognition Test. Further effect size analysis using semipartial correlation shows a weak correlation (*r* = .072). Similarly, the Picture Vocabulary Test demonstrated a significant correlation with the size of the left FAT's inferior coverage area (*p* = .0026). The corresponding effect size analysis also shows a weak correlation (*r* = .088). Moreover, we observed *p*‐values in the inferior coverage area of the right FAT approaching significance in both the Oral Reading Recognition Test (*p* = .071) and Picture Vocabulary Test (*p* = .068). The fractional anisotropy (FA) of the right FAT also showed a marginally significant correlation in the Picture Vocabulary Test (*p* = .056). Additionally, Picture Vocabulary shows a significant correlation with age (*p* = .0000011). This result is consistent with a study showing the increase in vocabulary size from 22 to 37 years old (Carroll et al., 2016).

Figure 6b shows the correlations with the two executive function tests, respectively. The results show significant correlations between the Card Sort Test score and age (*p* = .00015) and FA (*p* = .026). The effect size analysis showed a weak correlation between the FA of the right FAT and Card Sort Test score (*r* = .067). On the other hand, the Flanker test score is significantly correlated with age (*p* = .028) and sex (*p* = .00020). Notably, the coverage area of the both sides of FAT did not show a correlation with either test. In summary, our overall findings suggest a connection between the left FAT and language functions, and we did not identify a significant relationship between the FAT and executive functions.

### 3.5 Segmentation

**FIGURE 7:**
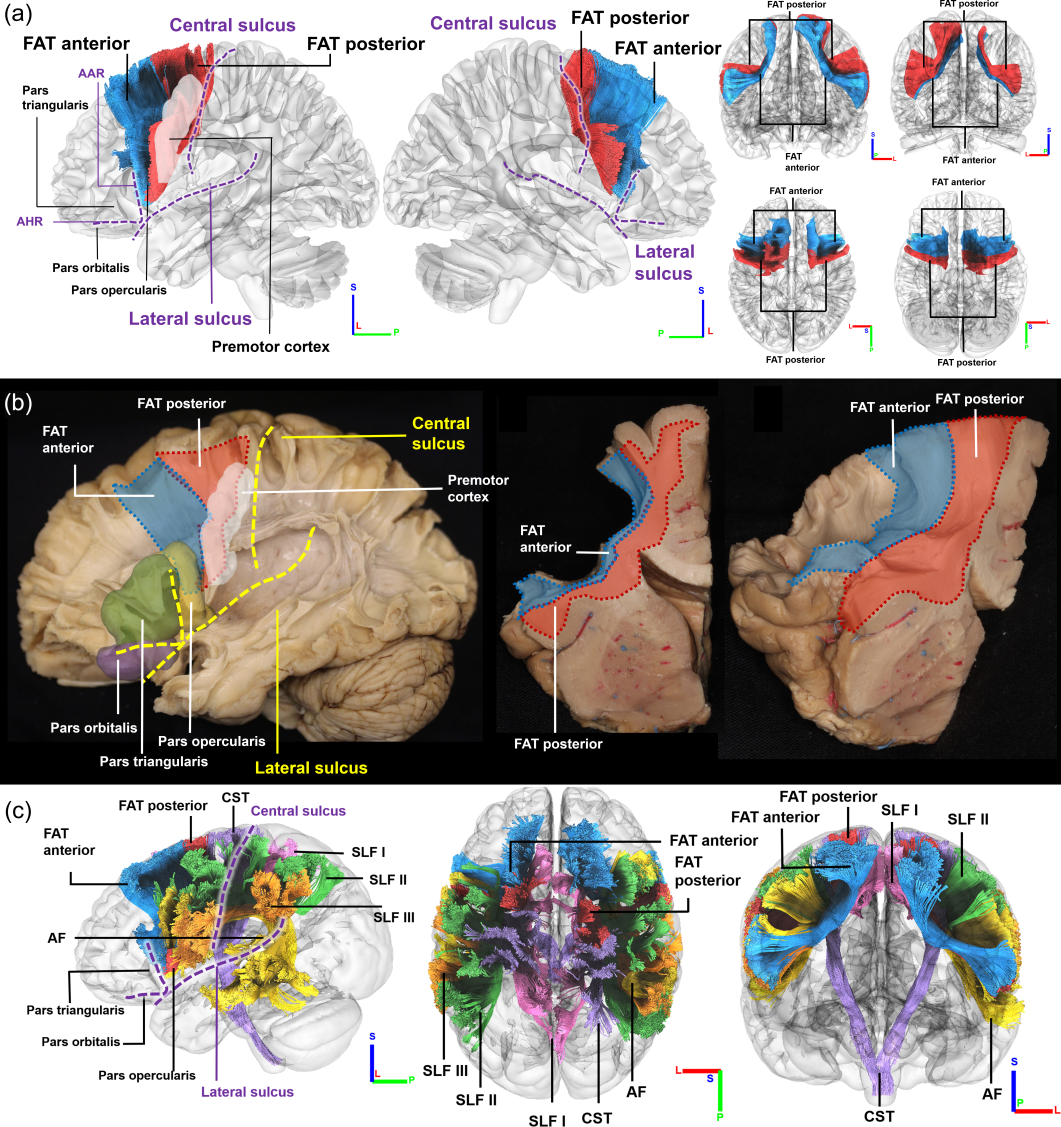
Segmentation of population‐averaged frontal aslant tract (FAT). (a) FAT can be segmented into the anterior FAT (blue) and posterior FAT (red). The posterior FAT projects to the inferior frontal gyrus (IFG), specifically to the pars opercularis. (b) Posterior FAT projects from the premotor cortex (white), whereas the anterior FAT projects to the pars opercularis (yellow), pars triangularis (green), and their anterior structures. (c) Compared to the anterior FAT, the posterior FAT has more areas interweaving with the surrounding pathways. The location of FAT runs more medial to other surrounding structures, such as superior longitudinal fasciculus (SLF) II (green), SLF III (red), and AF (yellow), whereas SLF I (pink) is medial to the FAT, and corticospinal tract (CST) (purple) is positioned at the posterior side of the FAT.

Following our previous analysis, the FAT could be segmented into the anterior (blue) and posterior FAT (red) based on their respective connecting regions, as shown in Figure 7a. The segmentation was based on the inferior frontal counterparts' connecting regions, since the superior area of the both bundles connect with SFG, lacking an obvious segmentation. Figure 7b anatomically depicts the curved shape of the FAT and its surrounding brain regions. Similar to the 3D model we created, the anterior FAT primarily projects to pars opercularis and partly pars triangularis, while the posterior FAT traverses premotor cortex. Figure 7c shows the white matter fiber pathways surrounding the FAT tracked using MRI data from an HCP young adult subject (#161731). The posterior FAT interweaves more with its surrounding white matter pathways compared to the anterior FAT, particularly with the SLF II and SLF III. The CST, shown in purple, is located posterior and runs parallel to the FAT, while the AF, SLF II, and SLF III run in the perpendicular direction and are more peripheral than the FAT.

## 4 DISCUSSION

In this study, we analyzed the individual differences of the FAT coverage in young adults using population probabilities and HCP‐MMP atlas to define the cortical regions. The findings indicated that there are large individual differences in the anterior FAT in the superior and middle frontal gyrus, and the superior part of the FAT has larger individual differences compared to the inferior part. The left FAT has more coverage than the right, and the distinction between the left and right is greater in the inferior and posterior side of the FAT coverage. The population probability of FAT coverage in cortical regions was also analyzed, revealing that the size of inferior coverage area is significantly correlated with Picture Vocabulary function. Furthermore, the left FAT inferior coverage area has a significant correlation with Picture Vocabulary function, indicating the structural and functional importance of the left FAT in language processing. On the other hand, FA, representing the anisotropy of FAT, shows no significant correlation with the functioning of FAT.

### 4.1 Individual differences

Our study is unique in that it is the first to analyze differences in FAT across a large cohort of 1065 subjects from the HCP. White matter tracts are known to have individual differences, and these differences may be attributed to genetic or environmental factors (Gu & Kanai, 2014). The structure of the FAT, which runs from the superior frontal gyrus to the IFG, has been previously identified by Catani et al. (2012). The original studies did not study its individual differences; however, our study found substantial individual differences in the FAT, which are not equally distributed. First, fewer individual differences are found in the inferior portion of the FAT. This could be related to the fact that FAT is funnel‐shaped and has less dispersion of the white matter tracts in the inferior coverage area. Previous studies have revealed that the FAT projects to pars opercularis or pars triangularis (Catani et al., 2012; Szmuda et al., 2017), while further studies indicated that FAT overlaps more with area pars opercularis than area pars triangularis (Garic et al., 2019; Szmuda et al., 2017). What we found is consistent: over 90% of the subjects had FAT connections in both pars opercularis and pars triangularis, indicating that most individuals have FAT connections in both areas. Furthermore, the population‐averaged FAT overlaps more with area pars opercularis than area pars triangularis. This finding highlights important implications for our understanding of language processing in the brain. The pars opercularis and pars triangularis are two regions of the frontal cortex that have been implicated in different aspects of language processing. An fMRI research revealed that the pars opercularis has been linked to syntactic processing, while the pars triangularis has been linked to semantic processing (Newman et al., 2003). The fact that the FAT overlaps more with the pars opercularis suggests that it may play a greater role in syntactic processing than semantic processing. This is consistent with previous research suggesting that the FAT is involved in the production and comprehension of semantic aspects of language (Dick et al., 2019; Friederici et al., 2003; Love et al., 2008). On the other hand, the fact that the overlap with the pars triangularis is lower suggests that the FAT may play a lesser role in syntactic processing. Understanding the specific neural circuits involved in different aspects of language processing may help to develop more targeted interventions for individuals with language disorders. Additionally, these findings may contribute to our understanding of the relationship between brain structure and function, and how individual differences in brain structure may relate to individual differences in language processing abilities.

Another key observation is our study is that the posterior FAT, which has less variability, connects the premotor cortex, whereas the anterior FAT, with greater variability, connects the IFG. This is an interesting finding and has potential implications for our understanding of the functional organization of the brain. The premotor cortex is involved in planning and executing motor movements, while the IFG is known to play a role in language processing and executive function (Purves, 2001). One possible explanation for the difference in variability between the anterior and posterior FAT is that the premotor cortex may be more genetically and developmentally constrained than the IFG. This could mean that there is less room for individual variability in the connections between the premotor cortex and posterior FAT.

Another possible explanation is that the anterior and posterior FAT play different roles in the functional organization of the brain. For instance, the anterior FAT may be more involved in language processing and executive function as well as inhibitory control, while the posterior FAT may be more involved in motor planning. Overall, different mechanisms underlying the development and organization of different regions of the brain may have implications for our understanding of individual differences in brain structure and function. Further research is needed to better understand the functional implications of the variability in the connections between the FAT and different regions of the brain, and how this variability relates to individual differences in cognitive and motor function.

### 4.2 Structural lateralization

In our study, our analysis showed significant left‐lateralization of the FAT. Our population analysis showed that the left FAT is in average larger than the right side, and it also has larger individual differences, specifically at the inferior part of the FAT. The structural lateralization has implications for our understanding of brain asymmetry and its relationship to cognitive function. Asymmetry in brain structure and function has been recognized as an important aspect of human cognition (O'Muircheartaigh et al., 2013), and understanding the neural mechanisms underlying this asymmetry can help us better understand how the brain works. The fact that the left FAT is larger and has more individual differences, particularly in the inferior part, may suggest that it is more involved in language‐related processes, such as semantic processing or word retrieval, than the right FAT. However, it is important to note that the superior portion of the FAT, which stems from the pre‐SMA and SMA, did not show as much left–right difference as the inferior portion. This suggests that the role of the superior portion of the FAT may not be as strongly lateralized as the inferior portion. Further research is needed to determine the specific functional roles of the superior and inferior portions of the FAT, and how these roles relate to lateralization and individual differences.

### 4.3 Structure–function correlation

One explanation for the structural lateralization of the FAT is that it reflects functional specialization in the brain. Our structure–function analysis showed that the inferior coverage area of the left FAT is significantly correlated with the performance of both of the language tests, namely the Oral Reading Recognition Test and the Picture Vocabulary Test. Notably, as a semantic test, the association between the Picture Vocabulary Test and the FAT was previously examined by Catani et al. (2013), and their findings indicated no significant relationship based on the number of streamlines for the tract, FA, and radial diffusivity. They did not study the topology of FAT and its correlation with language functions. In comparison, our study showed marginally significant correlation with FA (*p* = .056), likely due to a much larger sample size (*n* = 1065 vs. *n* = 35). The inferior coverage area of the FAT shows a much stronger correlation. Our result is consistent with previous studies that have shown that the left FAT is associated with language functions (Chernoff et al., 2018; Dick et al., 2019).

The correlation can be explained using the GODIVA language model (Bohland et al., 2010), an extension of the DIVA model (Guenther, 2006). According to DIVA model, language generation begins in the frontal operculum, specifically the pars triangularis, responsible for creating the speech sound map. GODIVA model refines specialization before this stage, with word input from inferior prefrontal regions, including pars triangularis, involved in higher‐level semantic processing. Phonological content representation occurs in the left inferior frontal sulcus, and structural frame representation takes place in the pre‐SMA. The results of phonological content representation in the frontal operculum were transmitted to the motor cortex, mirroring the process in the original DIVA model. Additionally, the results of pre‐SMA's phonological content representation were sent to the SMA, processed through the basal ganglia's motor loop, and then transmitted to the motor cortex, influencing its function. In both of our language tests, the Picture Vocabulary Test, focusing on semantic processing associated with pars triangularis, and the Oral Reading Test, involving speech sound generation related to pars triangularis, provided reasonable explanations for their correlation with the left inferior area of the FAT. While the model did not explicitly detail the functions between pre‐SMA and frontal operculum, the connection facilitated by the FAT between these two areas might suggest a potential association between speech sound mapping and structural frame functions.

Regarding executive functions, previous studies have suggested that the right FAT is more closely associated with inhibitory control, a component of executive functions (Chernoff et al., 2018; Dick et al., 2019). Aron et al. (2016) explored the mechanism of inhibitory control involving the subthalamic nucleus (STN) and proposed two hypothetical models. In one model, the initiation of the stopping signal originated from the right IFG, connected to the inferior part of the right FAT, and the control signal was eventually transmitted to the premotor area, linked to the superior part of the FAT. While the model indicated that the process of signal transportation involves the STN, it may also imply the role of the right FAT in the inhibitory control process. Here, we investigated two executive function tests. The Dimensional Change Card Sort Test exhibited a significant correlation with FA of the right FAT, whereas the Flanker Inhibitory Control displayed no significant correlation with the demographics related to the FAT. Nonetheless, since most whiter matter pathways' anisotropy are correlated with each other due to crossing and overlapping region, uncertainty arises as to whether the correlation is unique to the FAT. The relationship between the FAT and executive function is not as readily discernible compared to its language function counterpart.

### 4.4 Role in neurosurgery

Our study has significant implications for the field of neurosurgery, particularly in the context of low‐grade gliomas located in the superior frontal gyrus, including the SMA and preSMA. These types of tumors are commonly found in young patients, underscoring the importance of considering the FAT during presurgical planning and intraoperative procedures when removing brain lesions in this area. A thorough understanding of the FAT's fiber predominance, orientation, and volume is crucial in the intraoperative setting to ensure a safe surgical approach and minimize the potential for unnecessary damage to the brain, thereby reducing the risk of long‐term complications.

Previous research has shed light on the significance of the left FAT in various conditions. For instance, a study employing direct axonal stimulation and postoperative tractography investigated the role of white matter tracts, specifically the left FAT, in stuttering (Kemerdere et al., 2016). The findings revealed that the left FAT plays a pivotal role in stuttering and may be involved in a cortico‐subcortical circuit responsible for speech motor control. Another study reported that a unilateral lesion encompassing the insular and the FAT resulted in bilateral paralysis of the facial‐lip‐pharyngeal–laryngeal musculature (Martino et al., 2012). Additionally, disruption of the fronto‐parietal and frontal cortico‐subcortical connectivity, including the FAT, was found to be correlated with long‐lasting impairments of executive functions. These insights are invaluable for surgical planning and predicting neuropsychological disorders in brain tumor surgery (Cochereau et al., 2020).

There is a general consensus among researchers that the left FAT is intimately involved in speech initiation. This aligns with the role of the posterior segment of the FAT, which connects the SMA and preSMA to the facial‐lip motor cortex, contributing to the initiation of speech. However, it is worth noting that our study did not specifically examine language initiation due to the specific language tests employed. Nonetheless, our correlational evidence suggests that FAT may play a role in the semantic/ventral stream of language processing. This seems to match the role of the anterior segment of FAT that connects the superior frontal lobe to the inferior frontal regions, including Broca's area, which is known for its involvement in language production and comprehension. Further research is warranted to explore and elucidate the distinct functions of the anterior and posterior segments of the FAT more comprehensively.

Importantly, our study revealed substantial individual differences in the left anterior FAT, highlighting the potential risk for language deficits following surgery in this region. Therefore, for lesions located in the left inferior frontal lobe, employing an individualized mapping of the FAT could prove beneficial in reducing postsurgical functional deficits and optimizing patient outcomes.

### 4.5 Limitation

However, several limitations still exist in our study. We cannot conduct a population‐based study using cadavers. The population studies only used imaging data from the HCP. Furthermore, tractography has errors, and the indistinct boundaries between white matter pathways in specific individuals would lead to an inferior distinction. False continuations could occur with SLF and other nearby pathways. Finally, we have not conducted a functional mapping in this study to examine our functional claim about anterior and posterior FAT. This could be done by fMRI or intraoperative functional mapping.

Although language‐related functions have been analyzed in this study, motor planning functions are still to be analyzed in the future. Language fluency is said to be associated with the FAT in former studies (Chernoff et al., 2018; Dick et al., 2019), which is also not performed in this study and needs further analyzation. Additionally, due to limitations in our datasets, inhibitory control tests were not conducted, making us unable to investigate this function. For future clinical applications, the FAT segmentation could inform possible eloquent area, especially the anterior FAT.

In addition to examining individual differences in the topological distribution of the FAT, future studies could focus on investigating how these differences develop over time. Research could explore whether there are developmental differences in the FAT between children and adults, as well as the environmental and genetic factors that may contribute to these differences. Further studies could also investigate how the FAT varies in individuals with neurodevelopmental disorders, such as autism or schizophrenia. Another potential area of investigation is the functional significance of the variations in the FAT, including how they relate to individual differences in cognitive and behavioral functions. Finally, studies could explore how the variations in the FAT relate to the structural and functional connectivity of other neural networks in the brain, and how these networks are affected in neurological and psychiatric disorders.

## 5 CONCLUSION

In conclusion, our study revealed the individual variety of FAT. Our findings indicated significant individual differences in the left FAT, primarily in the superior and middle frontal gyrus, with the superior region showing more variability than the inferior region. Additionally, we observed pronounced left lateralization of the individual differences in FAT, particularly in the inferior and posterior portions. The size of the left FAT's inferior coverage areas correlated with language test, underscoring the importance of the left FAT in language processing. Future research should include functional mapping, motor planning functions, developmental differences, related neurodevelopmental disorders, and the functional significance of variation.

## AUTHOR CONTRIBUTIONS

WJL and JBM performed the analyses and contributed to the writing of the manuscript. DFC, TJ, MN, RG, and YA conducted the cadaver dissection and provided critical review of the manuscript. JFM initiated the study, reviewed the manuscript, and provided guidance. FCY contributed to the writing of the manuscript and oversaw the study.

## CONFLICT OF INTEREST STATEMENT

The authors declare no competing interest in this study.

## Data Availability

The tract‐to‐region connectome, based on the HCP‐MMP parcellations from a previous study (Yeh, 2022), was utilized in our analysis. The HCP‐MMP in ICBM152 space was obtained from an asymmetrical and improved reconstruction version of MMP 1.0 MNI projections from NeuroVault (https://identifiers.org/neurovault.collection:1549). The file is further edited with DSI Studio and shared on DSI Studio website: http://dsi-studio.labsolver.org. By mapping the intersection between the white matter bundles and cortical regions at the voxel level, a binary tract‐to‐region connection matrix was generated. The aggregation of binary matrices across the 1065 subjects results for the population probability of the tract‐to‐region connection, which is the tract‐to‐region connectome (available at http://brain.labsolver.org).
